# Supplementary material for: Enhancing secure multi-group data sharing through integration of IPFS and hyperledger fabric
Source: PeerJ Comput Sci. 2024 Mar 29;10:e1962. doi: 10.7717/peerj-cs.1962 (PMC11041925; doi:10.7717/peerj-cs.1962)
Supplement: Supplemental Information 1 — The implemented code comprises three fundamental components: 1. hyperledger-fabric-contract-java-dataShare: This module constitutes the chain code, uploaded to Hyperledger Fabric. 2. QKsysytem: Representing the data sharing system, this component serves as an intuitive interface enabling users to visualize and manage operations effectively. 3. io.jboot: As the data transfer system, this module ensures secure and encrypted transmission of data among nodes within the network. [file peerj-cs-10-1962-s001.zip › QKsysytem/src/main/resources/html/system/data/add.html]

#include("/template/common/layout/\_page\_layout.html")
#@layout()
#define css()
#end
#define js()
#end
#define content()

*\**code

*\**code description

*\**Type code

*\**Type description

*\**Sort number

*\**status

#statusOption(io.jboot.admin.service.entity.status.system.DataStatus::me(), '1')

save
reset

#end
